# Supplementary material for: FLT3-ITD Expression as a Potential Biomarker for the Assessment of Treatment Response in Patients with Acute Myeloid Leukemia
Source: Cancers (Basel). 2022 Aug 19;14(16):4006. doi: 10.3390/cancers14164006 (PMC9406666; doi:10.3390/cancers14164006)
Supplement: Supplementary file 1 [file cancers-14-04006-s001.zip › cancers-1819063-supplementary.pdf]

Supplementary Material

Table S1. Demographical, clinical and genetic characteristics of the 46 patients of the diagnosis cohort. PN: patient number of diagnosis cohort. WBC: white blood cell count, Hb: hemoglobin, PLT: platelet count, F: female, M: male, NQ: non-quantifiable AR due to the absence of wild-type allele, NA: not available, NP: not performed, NK: normal karyotype, AK: altered karyotype, CK: complex karyotype, NM: no metaphases.

PN	Sex	Age	WBC ($x10^9$/L)	Hb (g/dL)	PLT ($x10^9$/L)	Blasts (%)	*FLT3-ITD AR*		*NPM1*	*IDH1*	*IDH2*	*CEBPA*	*DNMT3A*	Karyotype
							DNA	*cDNA*						
1	F	75	92.5	8.8	18	63	0.34	0.61	-	NP	NP	NP	NP	NK
2	M	45	202.4	13.4	88	70	0.87	0.39	-	NP	NP	NP	NP	NK
3	F	50	54.0	9.4	19	88	0.87	1.06	+	NP	NP	NP	NP	NK
4	M	71	66.0	8.1	68	36	0.33	0.31	-	NP	NP	NP	NP	NK
5	M	52	50.9	7.9	29	88	0.43	0.67	+	NP	NP	NP	NP	NK
6	F	27	16.3	8.3	65	95	0.25/0.56	0.22/0.27	-	NP	NP	NP	NP	CK
7	M	41	127.7	7.7	115	81	0.53	1.2	-	NP	NP	NP	NP	NM
8	F	32	57.0	8.5	94	82	0.05/0.7	0.1/0.6	+	NP	NP	NP	NP	NK
9	F	37	10.1	9.6	54	73	1.04/0.12	4.6/0.7	+	-	NP	NP	NP	NK
10	F	69	230.7	12.4	20	72	0.57	0.98	+	-	NP	NP	NP	NM
11	M	87	86.7	10.8	109	NA	0.89	0.9	+	+	NP	NP	NP	NM
12	M	55	151.6	13.3	34	94	0.14/8.31	NQ/NQ	+	-	NP	NP	NP	NK
13	M	70	60.6	10	55	54	1.03/5.11	NQ/NQ	-	-	NP	NP	NP	AK
14	F	65	37.2	8.1	36	60	0.19/0.2	0.4/0.4	+	-	NP	NP	NP	NK
15	F	91	1.1	10.6	46	46	0.24	0.25	-	-	-	-	NP	NM
16	F	31	153.3	6.8	37	84	0.9	1.01	-	-	-	NP	-	NM
17	F	36	32.6	9.5	60	71	0.29/1.26	0.43/2.26	-	-	-	NP	-	CK
18	M	77	222.6	7.8	85	96	0.04	0.06	+	-	-	NP	NP	NK
19	M	65	23.4	11.6	107	60	0.41	0.99	+	-	-	NP	-	NK
20	M	72	259.0	10.9	134	27	0.18	0.14	-	-	+	NP	-	NK
21	M	85	0.8	8.8	223	55	0.25	0.36	-	-	-	-	-	AK
22	F	59	212.5	7.6	84	86	1.18	3.1	+	-	-	-	+	NK
23	M	72	170.05	10.9	52	98	0.75	0.9	+	-	-	-	-	NM
24	F	67	28.2	8.9	66	45	0.15/0.92	0.21/0.92	+	-	-	-	-	NK
25	M	84	70.8	9.2	86	79	0.65/0.7	0.64/0.7	-	NP	NP	NP	NP	NM
26	F	19	236.4	11.2	44	97	9.47	13	+	-	+	-	-	NK
27	M	85	134.8	9.7	56	NA	0.3	0.22	+	-	-	-	-	NM
28	F	49	164.5	13.1	79	95	0.49/0.55	0.36/0.74	+	-	-	+	-	NK
29	F	90	83.7	9.7	50	48	0.05	0.036	+	-	-	-	-	AK
30	M	79	10.4	11.2	994	94	1.1	0.99	-	-	-	-	-	NK
31	M	32	9.8	8.7	244	42	0.29	0.62	-	-	-	+	-	NK
32	M	27	79.4	8.2	42	82	0.74/0.27	1.6/0.5	-	-	-	-	-	NK
33	M	49	110.9	11.5	69	93	0.11/0.85	0.12/0.83	+	-	-	-	+	NK
34	F	52	25.0	7.3	70	77	0.13	0.18	-	-	-	-	-	NK
35	M	28	115.4	12	113	94	0.86	0.9	-	-	-	-	-	AK
36	F	73	63.1	9.1	89	76	0.76	0.85	+	-	-	+	+	NK
37	M	71	1.5	12.4	53	44	0.3	1	+	-	-	-	+	NM
38	M	53	7.1	12.2	328	70	0.68	0.6	+	-	-	-	+	NK
39	M	63	211.0	9.5	246	92	0.94	0.6	-	-	-	+	-	NK
40	M	59	235.3	6.6	72	47	1.03	9.1	+	-	-	-	+	NK
41	F	65	42.3	8.5	24	95	0.5/1/5	0.6/0.7/12	-	-	-	-	+	NK
42	M	52	76.8	11.3	125	75	0.88	0.89	-	-	-	-	-	NK
43	M	37	115.1	8.2	223	90	0.68/0.13	0.75/0.14	+	-	-	-	+	NK

44	F	60	11.5	12.8	129	75	0.71	1	+	-	+	-	+	NK
45	M	74	17.7	9.5	164	97	0.11/0	0.11/0.01	+	+	-	-	-	NK
46	M	89	6.6	13	81	28	0.017	0.06	-	-	-	-	-	NK

Table S2. Comparison between DNA and cDNA *FLT3*-ITD mutation ratio during follow-up in the post-allo-HSCT cohort. *Patient who relapsed after allo-HSCT. †Day 30 after allo-HSCT sample and pre-relapse sample of patient 2 were the same. PN: patient number of allo-HSCT cohort. Allo-HSCT: allogeneic hematopoietic stem cell transplantation.

PN	DNA			cDNA		
	pre-HSCT	Day 30	Pre-relapse	pre-HSCT	Day 30	Pre-relapse
1*	0	0	0.03	0.93	0	0.14
2*	0	0*	0	0	0*	0
3*	0	0	0	0	0	0
4*	0	0	0	0.1	0	0.16
5*	0	0	0	0	0	0
6*	0.19	0	0.03	0.9	0	0.53
7*	0	0	0	0	0	0.03
8*	0	0	0	0.04	0	0.57
9*	0	0	0.02	0	0	0.28
10*	0	0	0.04	0	0	0.47
11*	0	0	0.02	0	0	0.24
12*	0	0	0	0	0	0.05
13	0	0	-	0.03	0	-
14	0	0	-	0.04	0	-
15	0	0	-	0.05	0	-
16	0.01/0.03	0	-	0	0	-
17	0	0	-	0	0	-
18	0	0	-	0	0	-
19	0	0	-	0	0	-
20	0	0	-	0	0	-
21	0	0	-	0	0	-
22	0	0	-	0	0	-
23	0	0	-	0	0	-
24	0	0	-	0	0	-
25	0	0	-	0	0	-
26	0	0	-	0	0	-
27	0	0	-	0	0	-
28	0	0	-	0	0	-
29	0	0	-	0	0	-
30	0	0	-	0	0	-
31	0	0	-	0	0	-
32	0	0	-	0	0	-
33	0	0	-	0	0	-
34	0	0	-	0	0	-
